# Novel Highly Potent and Selective Sigma1 Receptor Antagonists Effectively Block the Binge Eating Episode in Female Rats

**DOI:** 10.1021/acschemneuro.0c00456

**Published:** 2020-09-04

**Authors:** Carlo Cifani, Emanuela Micioni Di Bonaventura, Luca Botticelli, Fabio Del Bello, Gianfabio Giorgioni, Pegi Pavletić, Alessandro Piergentili, Wilma Quaglia, Alessandro Bonifazi, Dirk Schepmann, Bernhard Wünsch, Giulio Vistoli, Maria Vittoria Micioni Di Bonaventura

**Affiliations:** † School of Pharmacy, Pharmacology Unit, University of Camerino, Via Madonna delle Carceri 9, 62032 Camerino, Italy; ‡ School of Pharmacy, Medicinal Chemistry Unit, University of Camerino, Via S. Agostino 1, 62032 Camerino, Italy; § Institut für Pharmazeutische und Medizinische Chemie, Universität Münster, Corrensstraße 48, 48149 Münster, Germany; ∥ Department of Pharmaceutical Sciences, University of Milan, Via Mangiagalli 25, 20133 Milano, Italy

**Keywords:** Selective sigma1 ligands, binge eating episode, highly palatable food, open field test, forced swimming test

## Abstract

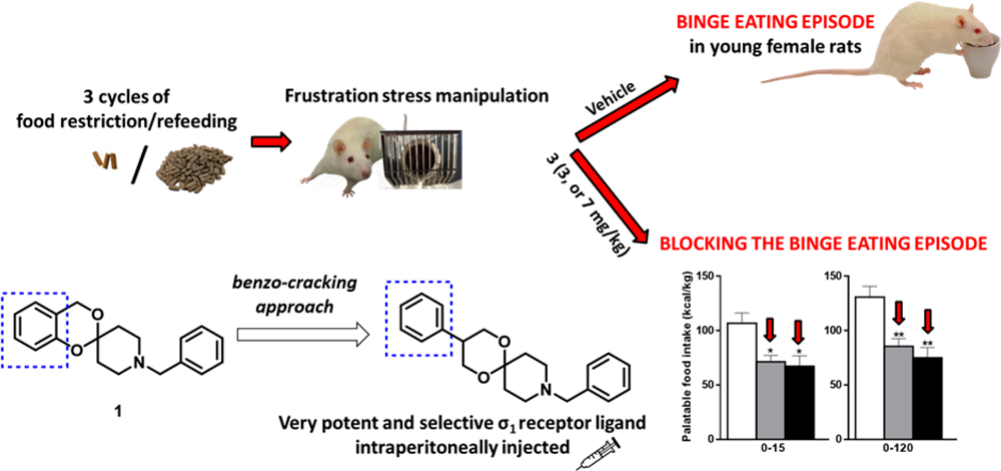

In this paper, the benzo-cracking approach was applied to the potent sigma1 (σ_1_) receptor antagonist **1** to afford the less conformationally constrained 1,3-dioxane derivatives **2** and **3**. To evaluate the effect of the increase in the distance between the two hydrophobic structural elements that flank the basic function, the *cis* and *trans* diastereomers of **4** and **5** were also prepared and studied. Compounds **2** and **3** showed affinity values at the σ_1_ receptor significantly higher than that of the lead compound **1**. In particular, **3** displayed unprecedented selectivity over the σ_2_ receptor, the phencyclidine site of the NMDA receptor, and opioid receptor subtypes, as well as over the dopamine transporter. Docking results supported the structure–activity relationship studies. Due to its interesting biological profile, derivative **3**, selected for an *in vivo* study in a validated preclinical model of binge eating, was able to counteract the overeating of palatable food only in binging rats, without affecting palatable food intake in the control group and anxiety-like and depression-related behaviors in female rats. This result strengthened the involvement of the σ_1_ receptor in the compulsive-like eating behavior and supported the σ_1_ receptor as a promising target for the management of eating disorders.

## Introduction

Sigma (σ) receptors are scarcely understood transmembrane proteins involved in a large number of cellular functions.^1^ Initially, they were classified as subtypes of the opioid receptor family, and subsequently, it was hypothesized that they corresponded to the phencyclidine (PCP) binding site of the ionotropic *N*-methyl-d-aspartate (NMDA) receptor. At present, they are reported as a distinctive receptor family, composed of two subtypes (σ_1_ and σ_2_ receptors).^1^ Both subtypes have been cloned,^2−5^ and the crystal structures of the σ_1_ receptor complexed with known agonists and antagonists have recently been reported.^6,7^ σ_1_ receptors work as molecular chaperones in the mitochondria-associated endoplasmic reticulum (ER) membrane and play a role in the cellular stress response and homeostasis.^8,9^

**Figure 1:**
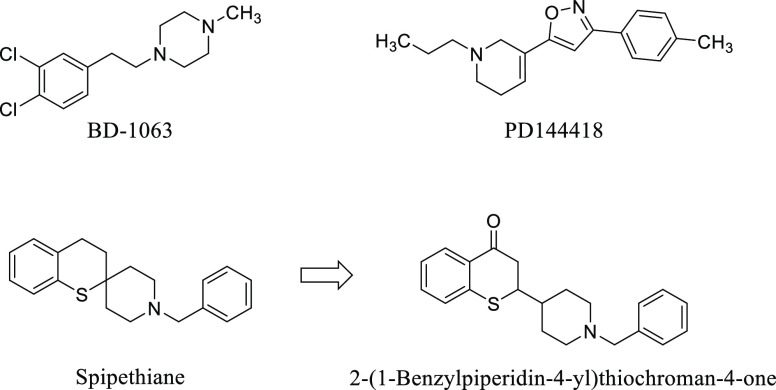
Structures of the σ_1_ antagonists BD-1063, PD144418, spipethiane, and 2-(1-benzylpiperidin-4-yl)thiochroman-4-one.

Their wide distribution in the nervous system and their involvement in several physiological and pathological conditions make σ_1_ receptors very promising targets for the management of numerous disorders. In particular, central σ_1_ receptors are implicated in different neuropsychiatric and neurodegenerative diseases^10−12^ as well as in pain.^13^ The observation that the σ_1_ agonist ANAVEX (NCT02244541) and the σ_1_ antagonist E-52862 (EudraCT number: 2012-000400-14) are being evaluated in clinical trials for the treatment of Alzheimer’s disease and neuropathic pain, respectively, supports the validity of σ_1_ receptors as clinical targets.^14^ Moreover, experimental evidence has demonstrated that the blockade of σ_1_ receptors can counteract the addictive effects elicited by psychostimulants^15,16^ and ethanol.^17−20^ While several papers report the involvement of σ_1_ receptors in drug abuse, very few studies suggest that this receptor system is implicated in binge eating behavior, despite many behavioral and brain mechanisms overlapping between food and drug addiction. In fact, compulsive fast overeating and strong craving, with a consequent withdrawal for hedonic food and impulsivity, are features correlated with binge eating behavior, similarly to substance dependence.^21,22^ In a pioneering study, the σ_1_ antagonist BD-1063 (Figure 1) was proven to reduce binge-like eating and to block compulsive eating in palatable rats, suggesting that the σ_1_ receptor system might play a role in binge-like eating following neurobiological adaptations.^23^ Moreover, a relationship between food-reinforced operant responding and σ_1_ receptors has recently been highlighted. Indeed, the potent σ_1_ antagonist PD144418 (Figure 1) was demonstrated to decrease the motivational effort of a food-reinforced behavior maintaining food palatability.^24^ Finally, in a recent study, we demonstrated that the spipethiane analogue 2-(1-benzylpiperidin-4-yl)thiochroman-4-one (Figure 1), behaving as a potent σ_1_ receptor antagonist,^25^ decreased the binge eating episode in female rats, supporting the involvement of σ_1_ receptors in compulsive-like eating disorder.^26^

**Figure 2:**
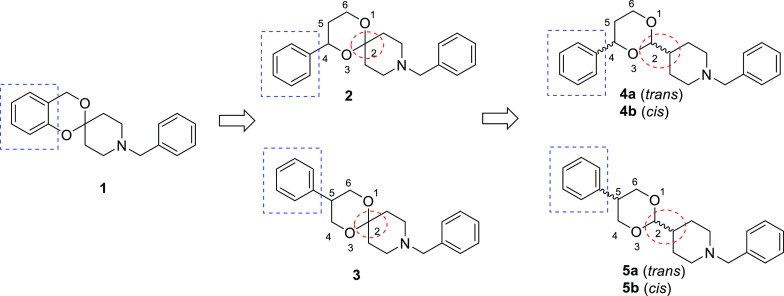
Structures of **2**–**5**, analogues of the potent σ_1_ ligand **1**.

Among the analogues of spipethiane, another potent σ_1_ receptor ligand (p*K*_i_ = 10.05), endowed with high σ_1_/σ_2_ selectivity (2515), is the 1,3-benzodioxane derivative **1** (Figure 2). Functional assays performed on MCF-7 and MCF-7/ADR highlighted the σ_1_ antagonist profile of this compound.^25^ With the aim to improve the σ_1_ receptor affinity and selectivity over σ_2_ subtype, the conformationally constrained 1,3-benzodioxane moiety of **1** was replaced by the more flexible 1,3-dioxane nucleus by the benzo-cracking approach.^27^ In particular, derivatives **2** and **3**, in which the phenyl substituent is linked to positions 4 and 5 of the 1,3-dioxane ring, respectively, were prepared and studied (Figure 2). Moreover, to evaluate the effect of the distance between the two hydrophobic portions that flank the basic function of **2** and **3**, the diastereomers **4a/b** and **5a/b** were also prepared and studied. In these novel derivatives, the *N*-benzylpiperidine moiety is spaced from the 1,3-dioxane ring (Figure 2), resulting in a further increase in the conformational flexibility of the molecule. The separation of the *cis* and *trans* diastereomers of **4** and **5** permitted us to evaluate the role played by the relative configuration on the σ_1_ receptor affinity.

The novel derivatives **2**–**5** were tested by radioligand binding assays at the σ_1_ and σ_2_ receptors. Moreover, to confirm the involvement of the σ_1_ receptor system in binge-like eating disorder, the aim of this work was also the evaluation of the most interesting compound **3** in a female rat model of binge eating. Finally, the affinities of compounds **2** and **3** were also assessed at the PCP site of the NMDA receptor, opioid receptors, and/or dopamine transporter (DAT), all of which play a role in binge eating disorders,^28^ considering that many σ_1_ ligands also bind these targets with high affinity.

## Results and Discussion

**Scheme 1:**
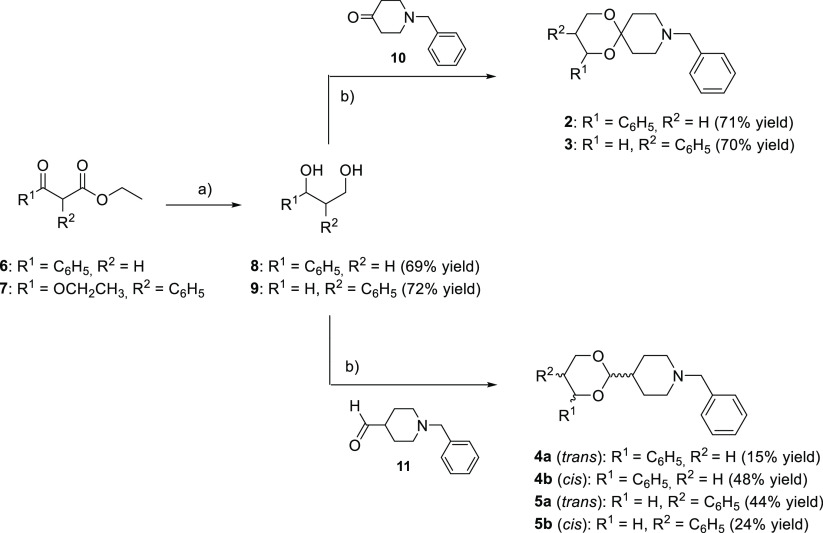
Conditions: (a) LiAlH_4_, Et_2_O, rt for 2 h; (b) *p*-toluenesulfonic acid, toluene, reflux for 5 h.

Derivatives **2**–**5** were synthesized following the synthetic route reported in Scheme 1.

The commercially available ethyl 3-oxo-3-phenylpropionate (**6**) and diethyl 2-phenylmalonate (**7**) were subjected to a reduction reaction with LiAlH_4_ to the corresponding diols **8** and **9**, respectively. The condensation of **8** and **9** with the suitable *N*-benzylpiperidine carbonyl derivatives **10** and **11** in the presence of *p*-toluenesulfonic acid afforded the desired derivatives **2** and **3** and the mixtures of the diastereomers **4a/b** and **5a/b**, respectively. The *cis* and *trans* diastereomers of **4** and **5** were separated by flash chromatography.

**Figure 3:**
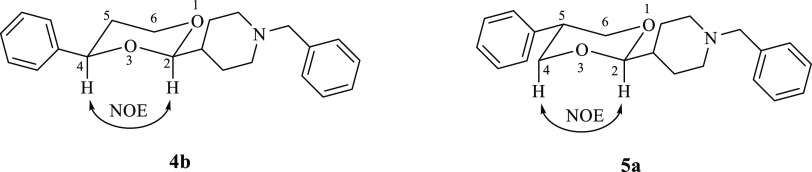
Structures of compounds **4b** and **5a**.

The stereochemical relationship between the *N*-benzylpiperidine moiety in position 2 and the phenyl substituent in positions 4 and 5 of **4a**/**b** and **5a**/**b**, respectively, was determined by ^1^H NMR analysis (NOESY studies). In particular, an evident nuclear Overhauser effect (NOE) was observed between the protons in positions 2 and 4 (4.48 and 4.65 ppm, respectively) of **4b**, highlighting that both the piperidine and phenyl rings in positions 2 and 4, respectively, are equatorially oriented. Therefore, the stereochemical relationship between the substituents in positions 2 and 4 is *cis* in **4b** and, consequently, *trans* in **4a** (Figure 3). Concerning **5a**, the axial proton in position 4 (δ 3.78 ppm) showed two large coupling constants (*J* = 10.8 Hz and *J* = 11.3 Hz), one with the geminal equatorial proton and the other with the axial proton in position 5. Consequently, the phenyl ring adopts an equatorial orientation. Moreover, a clear NOE was observed between the axial protons in positions 2 and 4 at 4.36 and 3.78 ppm, respectively, evidencing that the *N*-benzylpiperidine moiety also adopts an equatorial orientation. Therefore, the relative configuration between the substituents in positions 2 and 5 is *trans* in **5a** and, consequently, *cis* in **5b** (Figure 3).

The affinities of compounds **2**–**5** for σ_1_ and σ_2_ receptors were assessed on guinea pig brain and rat liver membranes, respectively. [^3^H]-(+)-pentazocine and [^3^H]-di-*o*-tolylguanidine in the presence of an excess of (+)-pentazocine were used as radioligands for σ_1_ and σ_2_ receptors, respectively.^29,30^ The p*K*_i_ values are reported in Table 1. The lead compound **1** was included for useful comparison.

**Table 1:**
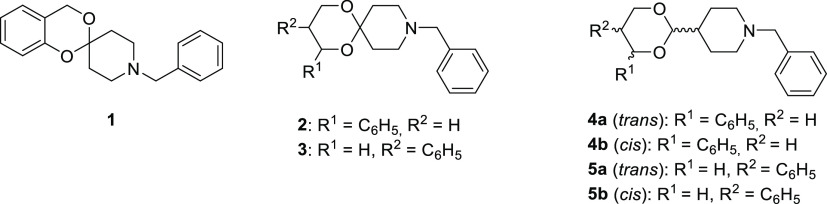
Affinity Values (p*K*_i_) of **1**–**5** at σ_1_ and σ_2_ Receptors and of **2** and **3** at DAT, the PCP Site of the NMDA Receptor, and μ, κ, and δ Opioid Receptor Subtypes^a^

	p*K*_i_						
compd	σ_1_	σ_2_	DAT	NMDA	μ	κ	δ
**1**	10.05 ± 0.08	6.65 ± 0.09					
**2**	11.00 ± 0.07	6.33 ± 0.11	<5	<5	<5	<5	8.60 ± 0.14
**3**	10.89 ± 0.05	6.09 ± 0.07	5.63 ± 0.09	<5	<5	<5	5.82 ± 0.08
**4a**	8.43 ± 0.07	6.75 ± 0.10					
**4b**	9.62 ± 0.15	7.42 ± 0.08					
**5a**	8.44 ± 0.14	7.25 ± 0.02					
**5b**	8.31 ± 0.06	6.60 ± 0.10					

a Equilibrium dissociation constants (*K*_i_) were derived from IC_50_ values using the Cheng–Prusoff equation.^34^ The reported p*K*_i_ values are the mean ± SEM of three to five independent experiments, each performed in triplicate, according to the methods described in the Supporting Information.

Compounds **2** and **3** were also evaluated for their affinity for DAT, the PCP site of the NMDA receptor, as well as μ, κ, and δ opioid receptor subtypes. The assays were performed with rat striatal ([^3^H]-WIN35,428), pig brain cortex ([^3^H]-(+)-MK-801), guinea pig brain ([^3^H]-DAMGO), guinea pig brain ([^3^H]-U-69593), and rat brain ([^3^H]-DPDPE) membranes for DAT, NMDA, and μ, κ, and δ opioid receptors, respectively.^29,31−33^ The p*K*_i_ values are shown in Table 1.

The data reported in Table 1 reveal that the benzo-cracking approach performed on the 1,3-benzodioxane derivative **1** is favorable for binding to the σ_1_ receptor, while it causes a slight reduction in σ_2_ receptor affinity, with a consequent increase in σ_1_/σ_2_ selectivity. In fact, both compounds **2** and **3** display very high affinity for σ_1_ receptor and remarkable σ_1_/σ_2_ selectivity. Several potent σ_1_ ligands belonging to different chemical classes and being highly selective over σ_2_ receptor have been discovered.^35^ Interestingly, **3** shows an impressive σ_1_/σ_2_ selectivity ratio (σ_1_/σ_2_ = 63 096) and, to our knowledge, is the most selective σ_1_ ligand reported so far. A significant reduction in affinity for the σ_1_ receptor and an increase in that for the σ_2_ receptor are observed when the benzo-cracking approach is combined with a further increase in the distance between the two lipophilic moieties of **2** and **3** (compounds **4a/b** and **5a/b**, respectively). Consequently, the σ_1_/σ_2_ affinity ratios displayed by **4a/b** and **5a/b** are significantly lower than those of **2** and **3**. Probably, the increase in the conformational freedom and in the distance between the two lipophilic portions is detrimental for the optimal interaction with σ_1_ receptor. Stereochemistry appears to play a role in the binding to the σ_1_ receptor when the phenyl ring is in position 4 of the 1,3-dioxane nucleus, with the *cis* isomer **4b** showing an affinity value significantly higher than that of the *trans* diastereomer **4a**. On the contrary, the *trans* and *cis* 5-phenyl diastereomers **5a** and **5b** show similar affinity values.

From the results obtained with the off-targets, it emerges that ligand **2** shows negligible affinity for DAT, NMDA, and μ and κ opioid receptors and high affinity for the δ subtype (p*K*_i_ = 8.60), although it is 251-fold lower than that for σ_1_. Interestingly, compound **3**, which also binds the δ receptor with submicromolar affinity, displays a remarkable selectivity for the σ_1_ receptor over all the evaluated targets (σ_1_/DAT = 181970, σ_1_/NMDA > 776247, σ_1_/μ > 776247, σ_1_/κ > 776247, σ_1_/δ = 117490). The binding profile of **3** is noticeable, given that many potent σ_1_ ligands also bind to DAT, NMDA, and/or opioid receptors with high affinity.^1,36,37^

**Figure 4:**
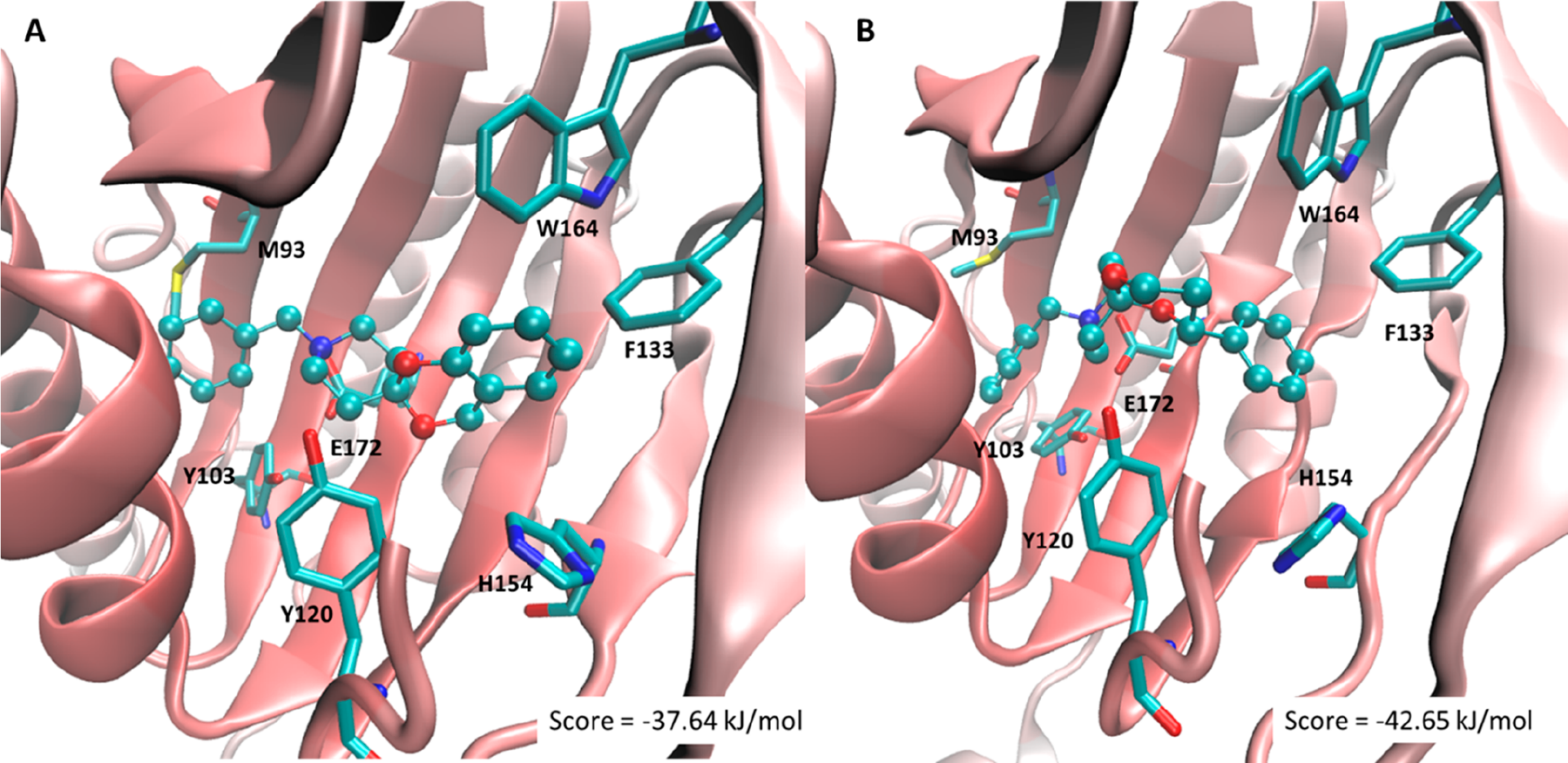
Main interactions stabilizing the putative complexes for **1** (A) and (*R*)-**2** (B) as computed using the resolved σ_1_ receptor structure. The reported scores are calculated by using the APBS method.

To rationalize the affinity profiles of the proposed ligands at the σ_1_ receptor, docking simulations were performed based on the resolved σ_1_ structure (PDB ID: 5HK1) using the PLANTS software and following the same recently reported computational protocol.^26^ As discussed below, the complex stability is evaluated by calculating the APBS score which is focused on the polar interactions.^38^Figure 4 compares the computed putative poses for **1** (Figure 4A) and **2** (Figure 4B) and reveals some differences which can justify the increase of affinity observed for the latter.

In detail, Figure 4A highlights the key interactions stabilized by **1** which can be schematized as follows: (a) the ligand ammonium head stabilizes a clear ion-pair with Glu172 reinforced by a H-bond with Tyr103; (b) the benzyl moiety is inserted within a hydrophobic subpocket where it mostly contacts alkyl side-chains plus π–π stacking with Tyr103 and a π-sulfur contact with Met93; (c) the benzodioxane system is accommodated within a subpocket lined by several aromatic residues while the O1 oxygen atom is engaged by a H-bond with Tyr120. The enantiomers of **2** afford very similar putative complexes, and attention is here focused on the complex for (*R*)-**2** since it shows a slightly better APBS score compared to (*S*)-**2** (−42.56 vs −41.38 kJ/mol). Specifically, Figure 4B emphasizes that (*R*)-**2** elicits an interaction pattern very similar to that already seen in Figure 4A, even though some key interactions appear to be enhanced when compared to those elicited by **1**. This positive effect can be seen in the contacts stabilized by (a) the benzyl moiety which elicits an optimized π–π stacking with Tyr103; (b) the dioxane oxygen atoms which better approach Tyr120; and (c) the phenyl ring which is engaged by an extended set of π–π stacking interactions with Phe107, Phe133, His154, and Trp164. These reinforced contacts are reflected into better complex stability as encoded by the scores displayed in Figure 4A. As also confirmed by its APBS score (−38.91 kJ/mol), compound **3** yields an in-between docking result, with the two aromatic rings being engaged by enhanced contacts, while the dioxane ring is unable to conveniently approach Tyr120, as seen in Figure 4B. Finally, compounds **4** and **5** reveal computed poses rather similar to those observed for the previous ligands, even though the free dioxane ring assumes a rather different arrangement which hampers its interactions with Tyr120. The lack of this contact induces flipped poses of the most hindered ligands by which the dioxane ring approaches Tyr103.

**Figure 5:**
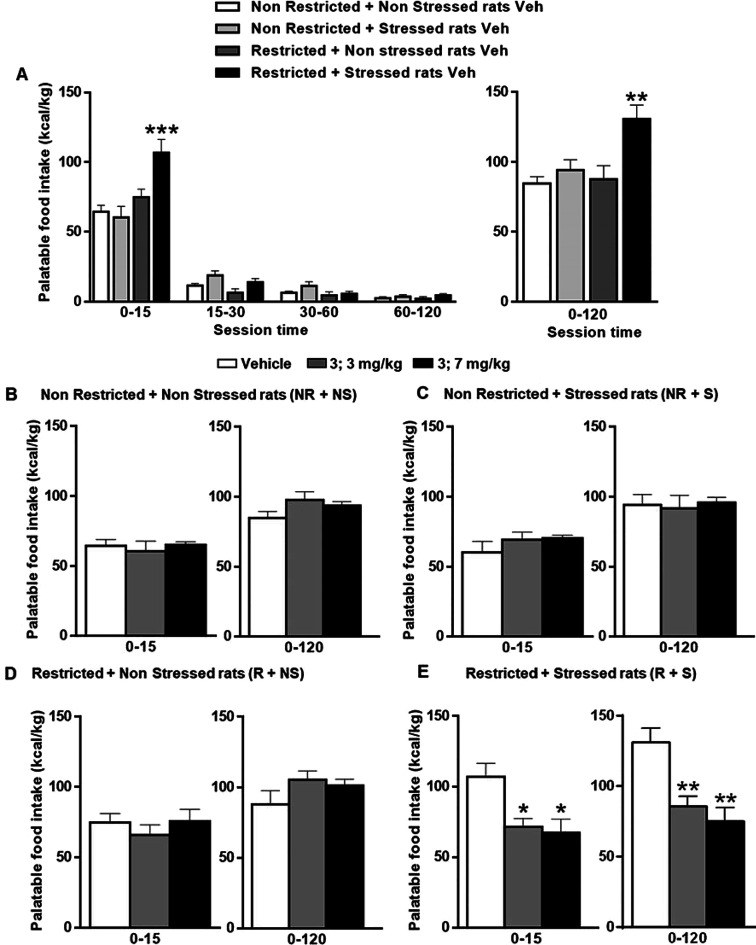
Administration of **3** blocked the episode of binge eating. (**A**) HPF intake shown in kcal/kg at different sessions time (0–15, 15–30, 30–60, 60–120 min; left) and at 120 min (right) in the vehicle (veh) injected rats. **P < 0.01; ***P < 0.001 different from the other three groups. (B) HPF eating (kcal/kg) after 15 min (left) or 120 min (right) to free access to cup containing chocolate paste in veh or treated rats: NR + NS (**B**, Non Restricted + Non Stressed), NR + S (**C**, Non Restricted + Stressed), R + NS (**D**, restricted + Non Stressed), R + S (**E**, Restricted + Stressed) groups. *P < 0.05; ***P < 0.01 vs R + S veh. Data are expressed as mean ± SEM, *N* = 6–8 per group.

Considering its intriguing σ_1_ affinity and selectivity profile, compound **3** was selected for the *in vivo* study, using a validated preclinical animal model of binge eating, to further investigate the function of the σ_1_ receptor system on a compulsive-like eating disorder. Female rats were used in relation to the higher prevalence of binge eating disorder and bulimia nervosa in women than in men.^39^ In the binge eating model,^40−42^ female rats were randomly separated into four groups: non restricted and not exposed to stress group (NR + NS); non restricted and exposed to stress group (NR + S); restricted and not exposed to stress group (R + NS); restricted and exposed to stress group (R + S). The association of three consecutive food restriction/refeeding periods and acute stress is able to trigger a strong increase of highly palatable food (HPF) consumption only in R + S rats in a short period of time (120 min). Stress is induced by placing a coffee cup containing HPF for 15 min, letting the animal see the cup and smell the HPF odor, without the possibility to eat it. Thus, on the binge test day, NR + NS and R + NS had immediate access to HPF for 120 min, whereas NR + S and R + S had free access to it only after 15 min of stress. This stressful condition, although mild, has been shown to enhance the corticosterone blood level in stressed rats.^43−46^ In line with our previous studies,^47,48^ the ANOVA in the vehicle groups revealed a marked interaction among the three factors (food restriction × stress × session time) [F_interaction_ (3,72) = 4.8; *P* < 0.01]. Bonferroni post hoc test revealed a significant (*P* < 0.01) increase in HPF consumption in the first 15 min in the R + S group (binging group), compared to the other three groups. On the other hand, during the time of the other sessions (15–30, 30–60, 60–120 min), no change in HPF intake was observed among all groups (Figure 5, left panel). At the end of the binge eating test (120 min), one-way ANOVA showed a two-way interaction (food restriction × stress) [F_interaction_ (1,24) = 4.3; *P* < 0.05] and the post hoc analyses (*P* < 0.01) revealed that only R + S rats significantly enhanced HPF eating with respect to the other rats (Figure 5, right panel). Thus, the stress exposure induced binge-like behavior only in previously restricted rats, which consumed a large amount of HPF within 15 min and no compensatory changes during the remaining 15–120 min were detected.

Acute intraperitoneal (i.p.) injection of **3**, 30 min before giving access to HPF, selectively blocked the episode of binge eating in a dose-dependent manner in the R + S group, without affecting consumption in the other experimental groups during 120 min of observation (Figure 5B–E).

Specifically in R + S rats, ANOVA reported a significant effect of treatment at 0–15 min [F(2,20) = 6.7; *P* < 0.05] and at 0–120 min [F(2,20) = 10.9; *P* < 0.01]. Post hoc analyses indicated that both dosages used (3 or 7 mg/kg) significantly decreased HPF consumption in R + S at each time point as indicated in Figure 5E.

In addition, to assess if the systemic injection of **3** may influence different aspects of animal behavior in the control or binging group, the open field (OF) test and forced swimming test (FST) were performed. The OF test is a validated test, commonly used for evaluating locomotor activity and anxiety-like behavior in rodents in an unfamiliar environment,^49^ while FST is a suitable tool for evaluating a depressed state.^50^ The administration of **3** was shown to not affect any measured behavioral parameters in these present tests. In fact, analyzing the locomotor activity in the entire OF arena, ANOVA showed a significant effect of restriction and stress conditions [F_restriction_ (1,48) = 7.9; *P* < 0.01; F_stress_ (1,48) = 30.9; *P* < 0.001] and no effect of the treatment with **3** [F_treatment_ (1,48) = 0.6; *P* > 0.05]. R + S veh and R + S **3** (7 mg/kg) showed the highest distance traveled compared to the other groups (Table 2).

**Table 2:** Behavioral Parameters in Female Rats Performing the Open Field and Forced Swimming Tests^a^

open field test								
	NR + NS		NR + S		R + NS		R + S	
parameters	veh	**3** (7 mg/kg)	veh	**3** (7 mg/kg)	veh	**3** (7 mg/kg)	veh	**3** (7 mg/kg)
tot. dist. trav. (cm)	2305.3 ± 357.3	2492.3 ± 225.3	3602.9 ± 269.3	3467.2 ± 411.8	2626.2 ± 242.5	3132.4 ± 331.6	4335.9 ± 419.3*	4461.5 ± 346.3*
tot. vert. counts	99 ± 4	93.7 ± 5	123.9 ± 6.1	125.9 ± 5.4	87 ± 3.3	94.3 ± 6.6	131.7 ± 4.3	126.6 ± 4.5
jump counts	104.6 ± 4.8	113 ± 17.1	137.1 ± 2.1	152 ± 32.5	97.7 ± 6.3	119.5 ± 9.1	172 ± 20.6	170.4 ± 19
stereot. counts	2367.1 ± 116.9	2500.3 ± 89.3	2477.4 ± 161.4	2270.4 ± 137.5	2030.5 ± 280.8	2516.7 ± 79.2	2305.1 ± 79.7	2401 ± 119.6
cent. dist. trav. (cm)	101.2 ± 25.6	112.7 ± 7.1	140.3 ± 30.1	148.1 ± 13.1	84.3 ± 17.1	96.4 ± 6	179.9 ± 24.8	177.5 ± 37.5
cent. zone entries	26.4 ± 4.6	28.1 ± 2.7	34.5 ± 4.1	34 ± 6.8	18.7 ± 2.8	22.5 ± 2.1	50.4 ± 2.4	47.2 ± 10

forced swimming test								
	NR + NS		NR + S		R + NS		R + S	
parameters	veh	**3** (7 mg/kg)	veh	**3** (7 mg/kg)	veh	**3** (7 mg/kg)	veh	**3** (7 mg/kg)
immobility time (s)	100.4 ± 12.94	106.7 ± 11.2	92.9 ± 13.5	111.1 ± 11.2	99.6 ± 10.4	94.8 ± 9.2	158.6 ± 10*	163.5 ± 22.5*

a In the entire open field arena: tot. dist. trav. (cm), total distance traveled; tot. vert. counts, total vertical counts; jump counts; stereot. counts, stereotypic counts. In the central zone of the open field box: cent. dist. trav. (cm), distance travelled in the center; cent. zone entries, number of entrances in the central zone. Data are the mean ± SEM. *p < 0.05 vs the other groups. *N* = 6–8 per group.

Regarding the other parameters, jump and total vertical counts were significantly affected only by stress [F_stress_ (1,48) = 11.9; *P* < 0.01] and [F_stress_ (1,48) = 86.7; *P* < 0.001], respectively, but not by restriction or treatment conditions. As shown in Table 2, the stress procedure appeared to increase the general arousal and this effect was confirmed by the significant gain in vehicle or treated stressed rats (NR + S and R + S) on distance traveled in the central zone [F_stress_ (1,48) = 19.7; *P* < 0.001] and on zone entries [F_stress_ (1,48) = 39.2; *P* < 0.001] into the central zone. In particular, the latest finding also suggested that stress does not influence anxiety-like behavior in stressed rats. Notably, the reduction of distance traveled or low numbers of entries into the central zone of the OF marked an increased emotionality and anxiety in rodents.^51^

Finally, no difference in stereotypic counts was found among the groups [F_restriction_ (1,48) = 0,7; *P* > 0.05; F_stress_ (1,48) = 0.02; *P* > 0.05; F_treatment_ (1,48) = 1.6; *P* > 0.05]. In addition, using the FST, ANOVA revealed that the immobility time was significantly impacted by restriction [F_restriction_ (1,49) = 8,2; *P* < 0.01], stress [F_stress_ (1,49) = 11.5; *P* < 0.01] and by the interaction between these two factors [F_interaction_ (1,49) = 12.7, *P* < 0.01], while compound **3** [F_treatment_ (1,48) = 1.6; *P* > 0.05] did not change the swimming/floating behavior. Post hoc tests exhibited a significantly longer immobility time in vehicle or treated R + S rats compared with the other groups, revealing that the cycle of food restriction plus stress may increase depression-like behaviors in female rats.

In summary, the stressed rats, particularly R + S, showed an increase of spontaneous locomotor and exploratory activity, including the central zone of the OF test, and the vehicle binging rats revealed the longest immobility time in the FST. In this context, **3** pretreatment did not impact the anxiety and depression-like behaviors in the control groups (NR + NS or NR + S or R + NS) and did not alter the emotional state detected in the binging rats.

## Conclusions

The replacement of the conformationally constrained 1,3-benzodioxane structure of **1** with the more flexible 1,3-dioxane ring by benzo-cracking approach led to derivatives **2** and **3**, which show very high affinity for σ_1_ receptor and a remarkable selectivity over σ_2_ subtype. Docking studies rationalized the affinity profiles of the proposed ligands on the σ_1_ receptor and gave useful information about the binding mode of this class of compounds. Showing significant affinity also for δ opioid subtype, **2** might be considered a dual σ_1_/δ receptor ligand. Interestingly, compound **3** displays an uncommon selectivity for the σ_1_ receptor over all the other evaluated targets. In *in vivo* studies, it was able to counteract the overeating of HPF only in binging rats, without affecting HPF intake in the control group and anxiety-like and depression-related behaviors in female rats. These findings reinforce the potential use of σ_1_ receptor antagonism to selectively block compulsive eating in binging rats, suggesting σ_1_ receptor antagonists as promising candidates to treat the binge episode, and are noteworthy, considering that, at present, the treatment approaches to manage pathological feeding behavior are limited.

## Methods

### Chemistry

#### General

Instruments used for the synthesis and characterization of compounds **2**–**9** are reported in the Supporting Information.

#### 9-Benzyl-2-phenyl-1,5-dioxa-9-azaspiro[5.5]undecane (**2**)

A mixture of **8** (1.7 g, 11.16 mmol), **10** (2.11 g, 11.16 mmol), and *p*-toluenesulfonic acid (0.85 g, 4.85) in toluene (50 mL) was heated at reflux for 5 h. After the mixture was cooled, water was added. The aqueous phase was basified with 2 N NaOH and extracted three times with CHCl_3_. The organic phase was dried (Na_2_SO_4_) and evaporated. The residue was purified by flash chromatography. Eluting with cyclohexane/EtOAc (7:3) afforded an oil (71% yield). ^1^H NMR (CDCl_3_) δ 1.61–2.60 (m, 10H, C*H*_2_ and piperidine), 3.51 (s, 2H, NC*H*_2_Ar), 3.90 (m, 1H, C*H*_*2*_O), 4.13 (m, 1H, C*H*_*2*_O), 4.98 (dd, 1H, ArC*H*O), 7.21–7.42 (m, 10H, Ar*H*). ESI/MS: *m*/*z* 324.2 [M + H]^+^. The free base was transformed into the oxalate salt that was crystallized from EtOH: mp 202–204 °C. Anal. Calcd for C_21_H_25_NO_2_·H_2_C_2_O_4_: C, 66.81%; H, 6.58%; N, 3.39%. Found: C, 67.05%; H, 6.42%; N, 3.50%.

#### 9-Benzyl-3-phenyl-1,5-dioxa-9-azaspiro[5.5]undecane (**3**)

This compound was synthesized from **9** and **10** according to the procedure described for **2**: an oil was obtained (70% yield). ^1^H NMR (CDCl_3_) δ 1.82 (m, 2H, piperidine), 2.18 (m, 2H, piperidine), 2.50 (m, 4H, piperidine), 3.18 (m, 1H, C*H*Ar), 3.53 (s, 2H, NC*H*_2_Ar), 3.99 (m, 4H, 2 × C*H*_*2*_O), 7.21–7.39 (m, 10H, Ar*H*). ESI/MS: *m*/*z* 324.2 [M + H]^+^. The free base was transformed into the oxalate salt that was crystallized from EtOH: mp 211–212 °C. Anal. Calcd for C_21_H_25_NO_2_·H_2_C_2_O_4_: C, 66.81%; H, 6.58%; N, 3.39%. Found: C, 66.59%; H, 6.40%; N, 3.19%.

#### 1-Benzyl-4-(4-phenyl-1,3-dioxan-2-yl)piperidine (**4**)

This compound was synthesized from **8** and **11** according to the procedure described for **2**, to give a mixture of the diastereomers **4a** and **4b**, that were separated by flash chromatography, eluting with cyclohexane/EtOAc (95:5).

The isomer **4a** eluted first as an oil (15% yield). ^1^H NMR (CDCl_3_) δ 1.28–2.42 (m, 9H, piperidine), 2.94 (m, 2H, piperidine), 3.49 (s, 2H, NC*H*_2_Ar), 3.92 (m, 1H, C*H*_*2*_O), 4.16 (m, 1H, C*H*_*2*_O), 4.42 (d, 1H, *J* = 6.5 Hz, OC*H*O), 5.19 (m, 1H, ArC*H*O), 7.20–7.42 (m, 10H, Ar*H*). ESI/MS: *m*/*z* 338.2 [M + H]^+^. The free base was transformed into the oxalate salt that was crystallized from 2-PrOH: mp 101–102 °C. Anal. Calcd for C_22_H_27_NO_2_·H_2_C_2_O_4_: C, 67.43%; H, 6.84%; N, 3.28%. Found: C, 67.27%, H, 6.96%; N, 3.50%.

The second fraction was the isomer **4b** (48% yield). ^1^H NMR (CDCl_3_) δ 1.19–1.94 (m, 9H, piperidine), 2.92 (m, 2H, piperidine), 3.50 (s, 2H, NC*H*_2_Ar), 3.89 (m, 1H, C*H*_*2*_O), 4.20 (m, 1H, C*H*_*2*_O), 4.48 (d, 1H, *J* = 5.6 Hz, OC*H*O), 4.65 (dd, 1H, *J* = 11.3, 2.3 Hz, ArC*H*O), 7.20–7.42 (m, 10H, Ar*H*). ESI/MS: *m*/*z* 338.2 [M + H]^+^. The free base was transformed into the oxalate salt that was crystallized from EtOH: mp 161–162 °C. Anal. Calcd for C_22_H_27_NO_2_·H_2_C_2_O_4_: C, 67.43%; H, 6.84%; N, 3.28%. Found: C, 67.70%, H, 6.98%; N, 3.05%.

#### 1-Benzyl-4-(5-phenyl-1,3-dioxan-2-yl)piperidine (**5**)

This compound was synthesized from **9** and **11** according to the procedure described for **2**, to give a mixture of the diastereomers **5a** and **5b**, that were separated by flash chromatography eluting with cyclohexane/EtOAc (95:5).

The isomer **5a** eluted first as an oil (44% yield). ^1^H NMR (CDCl_3_) δ 1.40–1.98 (m, 7H, piperidine), 2.88 (m, 2H, piperidine), 3.18 (m, 1H, C*H*Ar), 3.50 (s, 2H, NC*H*_2_Ar), 3.78 (dd, 1H, *J* = 11.3, 10.8 Hz, C*H*_*2*_O), 4.17 (dd, 1H, *J* = 11.3, 4.5 Hz, C*H*_*2*_O), 4.36 (d, 1H, *J* = 4.9 Hz, OC*H*O), 7.12–7.38 (m, 10H, Ar*H*). ESI/MS: *m*/*z* 338.2 [M + H]^+^. The free base was transformed into the oxalate salt that was crystallized from 2-PrOH: mp 158–160 °C. Anal. Calcd for C_22_H_27_NO_2_·H_2_C_2_O_4_: C, 67.43%; H, 6.84%; N, 3.28%. Found: C, 67.55%, H, 6.70%; N, 3.48%.

The second fraction was the isomer **5b** (24% yield). ^1^H NMR (CDCl_3_) δ 1.42–1.97 (m, 7H, piperidine), 2.61 (m, 1H, C*H*Ar), 2.92 (m, 2H, piperidine), 3.50 (s, 2H, NC*H*_2_Ar), 4.18 (m, 4H, 2 × C*H*_*2*_O), 4.42 (d, 1H, *J* = 5.2 Hz, OC*H*O), 7.18–7.59 (m, 10H, Ar*H*). ESI/MS: *m*/*z* 338.2 [M + H]^+^. The free base was transformed into the oxalate salt that was crystallized from 2-PrOH: mp 111–112 °C. Anal. Calcd for C_22_H_27_NO_2_·H_2_C_2_O_4_: C, 67.43%; H, 6.84%; N, 3.28%. Found: C, 67.61%, H, 6.97%; N, 3.41%.

#### 1-Phenylpropane-1,3-diol (**8**)

A solution of **6** (Aldrich) (1 g, 4.23 mmol) in dry Et_2_O (3 mL) was added dropwise to a suspension of LiAlH_4_ (0.17 g, 4.5 mmol) in dry Et_2_O (5 mL) at 0 °C under a nitrogen atmosphere. The mixture was stirred for 2 h at room temperature, then it was poured onto ice, and 2.5 M NaOH (12.65 mL) was added. After the precipitate was filtered off over Celite, the organic phase was dried (Na_2_SO_4_). The evaporation of the solvent afforded a residue that was purified by flash chromatography. Eluting with cycloexane/EtOAc (75:25) gave an oil (69% yield). ^1^H NMR (CDCl_3_) δ 1.86 (m, 2H, C*H*_*2*_), 3.24 (br s, 2H, exchangeable with D_2_O, 2 × O*H*), 3.79 (m, 2H, C*H*_*2*_O), 4.88 (dd, 1H, C*H*O), 7.25–7.36 (m, 5H, Ar*H*).

#### 2-Phenylpropane-1,3-diol (**9**)

This compound was synthesized from **7** (Aldrich) according to the procedure described for **8**: a white solid was obtained (72% yield). Mp 49–50 °C. ^1^H NMR (CDCl_3_) δ 1.95 (br s, 2H, exchangeable with D_2_O, 2 × O*H*), 3.08 (m, 1H, C*H*Ar), 3.96–4.03 (m, 4H, 2 × C*H*_*2*_O), 7.34–7.47 (m, 5H, Ar*H*).

### Radioligand Binding Studies

The experimental details of the binding studies at σ_1_, σ_2_, NMDA, opioid receptors, and DAT are reported in the Supporting Information.

### *In Vivo* studies

Female Sprague–Dawley rats (Charles River, Italy), 52 days old, were submitted to the binge eating protocol as described in previous works^52^ and in the Supporting Information.

The OF test was performed to evaluate locomotor activity, exploration, and anxiety-like behavior in rodents as described in previous studies.^53,54^ The FST is a validated tool, previously described^50^ to assess the depression-like behavior in rodents.

Compound **3** was dissolved in a 5% solution of DMSO in distilled water and administered i.p. (2 mL/kg) at 3 or 7 mg/kg doses. For the feeding test, **3** or the vehicle was injected 30 min before allowing access to HPF. For more detailed information, see the Supporting Information.

All rats in the estrous phase were excluded from the results, since binge eating episodes did not occur during this stage in female rats in the same animal model.^55−57^

## Supporting Information Available

Instruments used for the synthesis and characterization of compounds **2**–**9**, experimental details of the binding studies at σ_1_, σ_2_, NMDA, opioid receptors, and DAT, and binge eating experimental procedure (PDF)

## Abbreviations Used

PCP: phencyclidine
NMDA: *N*-methyl-d-aspartate
DAT: dopamine transporter
HPF: highly palatable food
i.p.: intraperitoneal
veh: vehicle
