# Supplementary material for: Novel Highly Potent and Selective Sigma1 Receptor Antagonists Effectively Block the Binge Eating Episode in Female Rats
Source: ACS Chem Neurosci. 2020 Sep 4;11(19):3107–16. doi: 10.1021/acschemneuro.0c00456 (PMC8011929; doi:10.1021/acschemneuro.0c00456)
Supplement: Supplementary file 1 — cn0c00456_si_001.pdf [file cn0c00456_si_001.pdf]

Supporting Information

Novel Highly Potent and Selective Sigma1 Receptor Antagonists Effectively Block the Binge Eating Episode in Female Rats

Carlo Cifani,[§] Emanuela Micioni Di Bonaventura,[§] Luca Botticelli,[§] Fabio Del Bello,[*,†] Gianfabio Giorgioni,[†] Pegi Pavletić,[†] Alessandro Piergentili,[†] Wilma Quaglia,[*,†] Alessandro Bonifazi,[†,‡] Dirk Schepmann,[⊥] Bernhard Wünsch,[⊥] Giulio Vistoli,[∥] and Maria Vittoria Micioni Di Bonaventura [§]

[§]*School of Pharmacy, Pharmacology Unit, University of Camerino, Via Madonna delle Carceri 9, 62032 Camerino, Italy*

[†]*School of Pharmacy, Medicinal Chemistry Unit, University of Camerino, Via S. Agostino 1, 62032 Camerino, Italy*

[‡]*Current Address: Medicinal Chemistry Section, Molecular Targets and Medications Discovery Branch, National Institute on Drug Abuse – Intramural Research Program, National Institutes of Health, Baltimore, Maryland, 333 Cassell Drive, Baltimore, Maryland 21224*

[⊥]*Institut für Pharmazeutische und Medizinische Chemie, Universität Münster, Correnssstraße 48, 48149 Münster, Germany*

[∥]*Department of Pharmaceutical Sciences, University of Milan, Via Mangiagalli 25, 20133 Milano, Italy*

INSTRUMENTS USED FOR THE SYNTHESIS AND CHARACTERIZATION OF COMPOUNDS 2-9

Melting points (mp) were taken in glass capillary tubes on a Büchi SMP-20 apparatus and are uncorrected. NMR spectra were recorded on either Varian Mercury AS400 or Bruker 500 MHz instruments and chemical shifts (ppm) are reported relative to tetramethylsilane. Spin multiplicities are given as s (singlet), d (doublet), dd (double doublet), t (triplet), or m (multiplet). IR spectra were recorded on PerkinElmer 297 instrument and spectral data (not shown because of the lack of unusual features) were obtained for all compounds reported and are consistent with the assigned structures. The microanalyses were recorded on FLASH 2000 instrument (ThermoFisher Scientific). The elemental composition of the compounds agreed to within ±0.4% of the calculated value. Mass spectra were obtained using a Hewlett Packard 1100 MSD instrument utilizing electron-spray ionization (ESI). All reactions were monitored by thin-layer chromatography (TLC) using silica gel plates (60 F254; Merck), visualizing with ultraviolet light. Chromatographic separations were performed on silica gel columns (Kieselgel 40, 0.040−0.063 mm, Merck) by flash chromatography. Compounds were named following IUPAC rules as applied by ChemBioDraw Ultra (version 11.0) software for systematically naming organic chemicals.

RADIOLIGAND BINDING ASSAYS DETAILS

Radioligand binding assays at the PCP site of NMDA receptor, DAT, σ_1 and σ_2 receptors and μ, κ, δ opioid receptors

Materials

The guinea pig brains, rat brains and rat liver for the σ_1, σ_2, μ-, κ- and δ-opioid receptor binding assays were commercially available (Harlan-Winkelmann, Borchen, Germany). The pig brains for the performance of the binding assay to the PCP-binding site of the NMDA receptor were a kind donation of the local slaughterhouse (Coesfeld, Germany). Homogenizers: Elvehjem Potter (B. Braun Biotech International, Melsungen, Germany) and Soniprep 150, MSE, London, UK). Centrifuges: Cooling

centrifuge model Rotina 35R (Hettich, Tuttlingen, Germany) and High-speed cooling centrifuge model Sorvall RC-5C plus (Thermo Fisher Scientific, Langenselbold, Germany). Multiplates: standard 96-well multiplates (Diagonal, Muenster, Germany). Shaker: self-made device with adjustable temperature and tumbling speed (scientific workshop of the institute). Harvester: MicroBeta FilterMate-96 Harvester. Filter: Printed Filtermat Typ A and B. Scintillator: Meltilex (Typ A or B) solid state scintillator. Scintillation analyzer: MicroBeta Trilux (all Perkin Elmer LAS, Rodgau-Jügesheim, Germany).

Preparation of membrane homogenates from pig brain cortex

Fresh pig brain cortex was homogenized with the potter (500-800 rpm, 10 up-and-down strokes) in 6 volumes of cold 0.32 M sucrose. The suspension was centrifuged at 1200 x g for 10 min at 4 °C. The supernatant was separated and centrifuged at 31,000 x g for 20 min at 4 °C. The pellet was resuspended in 5-6 volumes of TRIS/EDTA buffer (5 mM/1 mM, pH 7.5) and centrifuged again at 31,000 x g (20 min, 4 °C). The final pellet was resuspended in 5-6 volumes of buffer and frozen (−80 °C) in 1.5 mL portions containing about 0.8 mg protein/mL.

Preparation of membrane homogenates from guinea pig brain

5 guinea pig brains were homogenized with the potter (500-800 rpm, 10 up-and-down strokes) in 6 volumes of cold 0.32 M sucrose. The suspension was centrifuged at 1200 x *g* for 10 min at 4 °C. The supernatant was separated and centrifuged at 23500 x *g* for 20 min at 4 °C. The pellet was resuspended in 5-6 volumes of buffer (50 mM TRIS, pH 7.4) and centrifuged again at 23500 x g (20 min, 4 °C). This procedure was repeated twice. The final pellet was resuspended in 5-6 volumes of buffer and frozen (−80 °C) in 1.5 mL portions containing about 1.5 mg protein/mL.

Preparation of membrane homogenates from rat brain

5 rat brains (species: Sprague Dawley rats) were homogenized with the potter (500-800 rpm, 10 up-and-down strokes) in 6 volumes of cold 0.32 M sucrose. The suspension was centrifuged at 1200 x g for 10 min at 4 °C. The supernatant was separated and centrifuged at 23500 x g for 20 min at 4 °C. The pellet was resuspended in 5-6 volumes of buffer (50 mM TRIS, pH 7.4) and centrifuged again at

23500 x *g* (20 min, 4 °C). This procedure was repeated twice. The final pellet was resuspended in 5-6 volumes of buffer and frozen (−80 °C) in 1.5 mL portions containing about 1.5 mg protein/mL.

Preparation of membrane homogenates from rat liver

Two rat livers were cut into small pieces and homogenized with the potter (500-800 rpm, 10 up-and-down strokes) in 6 volumes of cold 0.32 M sucrose. The suspension was centrifuged at 1,200 x *g* for 10 min at 4 °C. The supernatant was separated and centrifuged at 31,000 x *g* for 20 min at 4 °C. The pellet was resuspended in 5-6 volumes of buffer (50 mM TRIS, pH 8.0) and incubated at room temperature for 30 min. After the incubation, the suspension was centrifuged again at 31,000 x *g* for 20 min at 4 °C. The final pellet was resuspended in 5-6 volumes of buffer and stored at -80,°C in 1.5 mL portions containing about 2 mg protein/mL.

Protein determination

The protein concentration was determined by the method of Bradford,[1] modified by Stoscheck.[2] The Bradford solution was prepared by dissolving 5 mg of Coomassie Brilliant Blue G 250 in 2.5 mL of EtOH (95 %, v/v). 10 mL deionized H_2O and 5 mL phosphoric acid (85%, m/v) were added to this solution, the mixture was stirred and filled to a total volume of 50.0 mL with deionized water. The calibration was carried out using bovine serum albumin as a standard in 9 concentrations (0.1, 0.2, 0.4, 0.6, 0.8, 1.0, 1.5, 2.0 and 4.0 mg /mL). In a 96-well standard multiplate, 10 µL of the calibration solution or 10 µL of the membrane receptor preparation were mixed with 190 µL of the Bradford solution, respectively. After 5 min, the UV absorption of the protein-dye complex at λ = 595 nm was measured with a platereader (Tecan Genios, Tecan, Crailsheim, Germany).

General procedures for the binding assays

The test compound solutions were prepared by dissolving approximately 10 µmol (usually 2-4 mg) of test compound in DMSO so that a 10 mM stock solution was obtained. To obtain the required test solutions for the assay, the DMSO stock solution was diluted with the respective assay buffer. The filtermats were presoaked in 0.5% aqueous polyethylenimine solution for 2 h at room temperature before use. All binding experiments were carried out in duplicates in the 96-well plates. The

concentrations given are the final concentration in the assay. Generally, the assays were performed by addition of 50 µL of the respective assay buffer, 50 µL test compound solution in various concentrations (10^{-5}, 10^{-6}, 10^{-7}, 10^{-8}, 10^{-9} and 10^{-10} mol/L), 50 µL of corresponding radioligand solution and 50 µL of the respective receptor preparation into each well of the multiplate (total volume 200 µL). The receptor preparation was always added last. During the incubation, the plates were shaken at a speed of 500-600 rpm at the specified temperature. Unless otherwise noted, the assays were terminated after 120 min by rapid filtration using the harvester. During the filtration each well was washed five times with 300 µL of water. Subsequently, the filtermats were dried at 95 °C. The solid scintillator was melted on the dried filtermats at a temperature of 95 °C for 5 minutes. After solidifying of the scintillator at room temperature, the trapped radioactivity in the filtermats was measured with the scintillation analyzer. Each position on the filtermat corresponding to one well of the multiplate was measured for 5 min with the [^{3}H]-counting protocol. The overall counting efficiency was 20%. The IC_{50}-values were calculated with the program GraphPad Prism® 3.0 (GraphPad Software, San Diego, CA, USA) by non-linear regression analysis. Subsequently, the IC_{50} values were transformed into K_i-values using the equation of Cheng and Prusoff. The K_i-values are given as mean value $\pm$ SEM from three independent experiments.

Performance of the binding assays

PCP binding site of the NMDA receptor

The assay was performed with the radioligand [^{3}H]-(+)-MK-801 (22.0 Ci/mmol; Perkin Elmer). The thawed membrane preparation of pig brain (about 100 µg of the protein) was incubated with various concentrations of test compounds, 2 nM [^{3}H]-(+)-MK-801, and TRIS/EDTA buffer (5 mM/1 mM, pH 7.5) at room temperature. The non-specific binding was determined with 10 µM unlabeled (+)-MK-801. The K_d-value of (+)-MK-801 is 2.26 nM.

σ_1 receptor

The assay was performed with the radioligand [^{3}H]-(+)-Pentazocine (22.0 Ci/mmol; Perkin Elmer). The thawed membrane preparation of guinea pig brain cortex (about 100 µg of the protein) was

incubated with various concentrations of test compounds, 2 nM [^{3}H]-(+)-Pentazocine, and TRIS buffer (50 mM, pH 7.4) at 37 °C. The non-specific binding was determined with 10 µM unlabeled (+)-Pentazocine. The K_d-value of (+)-Pentazocine is 2.9 nM.[3]

σ_2 receptor

The assays were performed with the radioligand [^{3}H]DTG (specific activity 50 Ci/mmol; ARC, St. Louis, MO, USA). The thawed membrane preparations (either membrane fragments prepared from approximately 200,000 RT-4 cells containing 150 µg protein or rat liver preparation containing 100 µg protein) were incubated with various concentrations of the test compound, 3 nM [^{3}H]DTG and buffer containing (+)-pentazocine (500 nM (+)-pentazocine in 50 mM TRIS, pH 8.0) at 37 °C (RT-4 cell fragments) or room temperature (rat liver membranes). The non-specific binding was determined with 10 µM non-labeled DTG. The K_d values are 8.3 nM (RT-4 cells) or 17.9 nM (rat liver).

κ opioid receptor

The assay was performed with the radioligand [^{3}H]-U-69593 (55 Ci/mmol, Amersham, Little Chalfont, UK). The thawed guinea pig brain membrane preparation (about 100 µg of the protein) was incubated with various concentrations of test compounds, 1 nM [^{3}H]-U-69593, and TRIS-$MgCl_2$-Puffer (50 mM, 8 mM $MgCl_2$, pH 7.4) at 37 °C. The non-specific binding was determined with 10 µM unlabeled U-69593. The K_d-value of U-69593 is 0.69 nM.

µ opioid receptor

The assay was performed with the radioligand [^{3}H]-DAMGO (51 Ci/mmol, Perkin Elmer LAS). The thawed guinea pig brain membrane preparation (about 100 µg of the protein) was incubated with various concentrations of test compounds, 3 nM [^{3}H]-DAMGO, and TRIS-$MgCl_2$-Puffer (50 mM, 8 mM $MgCl_2$, pH 7.4) at 37 °C. The non-specific binding was determined with 10 µM unlabeled Naloxon. The K_d-value of DAMGO is 0.57 nM.

human µ opioid receptor

HEK293 cells stably expressing hµOR were grown in a DMEM medium, supplemented with 10% FBS, 2 mM L-glutamine, 1% penicillin-streptomycin (or antibiotic/antimycotic) and hygromycin B

(50 μg/mL).[4] Upon reaching 80-90% confluence, cells were harvested using pre-mixed Earle's Balanced Salt Solution (EBSS) with 5 mM EDTA (Life Technologies) and centrifuged at 3,000 rpm for 10 min at 21 °C. The supernatant was removed, and the pellet was resuspended in 10 mL hypotonic lysis buffer (5 mM $MgCl_2$, 5 mM Tris, pH 7.4 at 4 °C) and centrifuged at 14,500 rpm (~25,000 g) for 30 min at 4 °C. The pellet was then resuspended in fresh binding buffer. A Bradford protein assay (Bio-Rad, Hercules, CA) was used to determine the protein concentration. The binding buffer was made of 50 mM Tris and 5 mM $MgCl_2$ at pH 7.4.[6] The experiments were performed in presence of [^{3}H]-DAMGO (final concentration 3 nM) and 30 μg/well of membranes (final concentration). The reactions were incubated for 60 min at RT and terminated by rapid filtration through Perkin Elmer Uni-Filter-96 GF/B, presoaked for 60 min in 0.5% polyethylenimine. The non-specific binding was determined using 10 μM C-TOP or cold DAMGO. The radioligand K_d (2.94 nM) was measured via radioligand saturation experiments.

δ opioid receptor

The assay was performed with the radioligand [^{3}H]-DPDPE (69 Ci/mmol, Amersham). The thawed rat membrane preparation (about 75 μg of the protein) was incubated with various concentrations of test compounds, 3 nM [^{3}H]-DPDPE, and TRIS-$MgCl_2$-PMSF-buffer (50 mM, 8 mM $MgCl_2$, 400 μM PMSF, pH 7.4) at 37 °C. The non-specific binding was determined with 10 μM unlabeled Morphine. The K_d-value of DPDPE is 0.65 nM.

DAT radioligand binding in rat striatum

Frozen brain striata dissected from male Sprague-Dawley rat brains (supplied in ice cold PBS buffer from BioreclamationIVT (Hicksville, NY)) were homogenized in 10-20 volumes (w/v) of modified sucrose phosphate buffer (0.32M Sucrose, 7.74 mM Na_2HPO_4, 2.26mM NaH_2PO_4 adjusted to pH 7.4 at 25 C) using a Brinkman Polytron (two cycles at setting 6 for 10 s each). The tissue was centrifuged at 20,000 rpm for 10 min at 4 ^{O}C. The pellet was suspended in cold buffer and centrifuged again.[5] The resulting pellet was resuspended in cold buffer at a concentration of 15 mg/mL OWW (original

wet weight). On test day, all test compounds were freshly diluted in 30% DMSO and 70% H_2O to a stock concentration of 1 mM or 100 µM. To assist the solubilization of free-base compounds, 10 µl of glacial acetic acid was added along with the DMSO (in place of 10 µl final H_2O volume). Each test compound was then diluted into 10 half-log serial dilutions using 30% DMSO vehicle. Radioligand competition experiments were conducted in 96-well plates containing 50 µL of diluted test compound, 300 µl of fresh binding buffer, 50 µl of radioligand diluted in binding buffer ([^{3}H]-WIN35,428: 1.5 nM final concentration; ARC, Saint Louis, MO) and 100 µl of tissue preparation (1.5 mg of brain striatum membranes per well). Aliquots of [^{3}H]-WIN35,428 solution were also quantified accurately to determine how much radioactivity was added. Non-specific binding was determined using 10 µL Indatraline and total binding was determined with 30% DMSO vehicle. The reaction was started with the addition of the tissue. All compound dilutions were tested in triplicate and the reaction incubated for 120 min at 4 ^{O}C. The reaction was terminated by filtration through Perkin Elmer Uni-Filter-96 GF/B, presoaked for 120 min in 0.05% polyethylenimine, using a Brandel 96-Well Plates Harvester Manifold (Brandel Instruments, Gaithersburg, MD). The filters were washed 3 times with 3 mL (3 x 1 mL/well) of ice cold binding buffer. 65 µL Perkin Elmer MicroScint20 Scintillation Cocktail was added to each well and filters were counted using a Perkin Elmer MicroBeta Microplate Counter (efficiency: 32.3%). IC_{50} values for each compound were determined from dose-response curves and K_i values were calculated using the Cheng-Prusoff equation. K_d value for [^{3}H]- WIN35,428 (28.1 nM) was determined via separate homologous competitive binding experiments. These analyses were performed using GraphPad Prism version 6.00 for Macintosh (GraphPad Software, San Diego, CA). K_i values were determined from at least 3 independent experiments and are reported as mean ± SEM.

BINGE EATING EXPERIMENTAL PROCEDURE

Female Sprague Dawley rats (Charles River, Calco, Lecco, Italy), 200–225 g at the beginning of the

experiments, were housed under a 12-h light/dark cycle (lights on at 9:00 a.m.), at constant temperature (20–22° C) and humidity (45–55%), and with access to food and water ad libitum for one week before the experiments. All experiments were carried out in accordance with the European directive 2010/63/UE governing animal welfare, and with the Italian Ministry of Health guidelines for the care and use of laboratory animals. Rats were fed with standard food pellets, 4RF18 (Mucedola, Milan, Italy), while the highly palatable food (HPF) used during the binge eating protocol[6] was a paste, offered in a china coffee cup, mixing: 52% Nutella (Ferrero, Torino, Italy) chocolate cream, 33% food pellets, and 15% water. Female rats were divided into four groups: non restricted and not exposed to stress group (NR + NS); non restricted and exposed to stress group (NR + S); restricted and not exposed to stress group (R + NS); restricted and exposed to stress group (R + S). Briefly, they were exposed (or not exposed) for 24 days to three 8 days cycles of food restriction/refeeding, during which all rats became familiar with the HPF. On the binge intake test day, NR + NS and R + NS had immediate access to the HPF for 120 min, whereas NR + S and R + S had free access to it only after 15 min of stress. During the stress procedure the rats could see and smell the palatable food inside a cup, but they could not access it for 15 min, engaging in repeated movements of the forepaws, head, and trunk to reach the HPF. After 15 min, the HPF cup was placed inside the cage, and food intake was determined for 2 h. We operationally define "binge eating episode" the significantly higher HPF consumption during the 2 h test in the R + S rats compared to the other experimental conditions.

Immediately after testing, we collected vaginal smears and analyzed them to assess the ovarian phase. We previously found that binge eating episode in the present animal model [7] is not observed during the estrous phase,[8, 9] thus all rats in this phase were excluded from the experiments.

Behavioral tests

Open field test was performed in all groups of rats to evaluate locomotor activity, exploration, and anxiety-like behavior [7], measuring the following activity parameters for 10 min: total distance

travelled; total vertical counts; jump counts; stereotypic counts and the number of entrances in the central zone. Automated locomotor activity boxes (square plastic boxes with a 43 x 43 cm arena and a 25 x 25 cm central zone; Med Associates, St Albans, Vermont, USA) were used to quantify spontaneous activity parameters. Locomotor activity was recorded automatically by interruption of two orthogonal light beams (3.5 and 13 cm above the activity box floor), which were connected to automatic software. Increased locomotor activity in the entire field was considered a sign of behavioral arousal; and reduced locomotor activity in the central zone and numbers of entries into the central zone were considered signs of increased emotionality, anxiety, or fear in rodents. [7, 10] Between test sessions, the apparatus was cleaned with alcohol (70%) and dried with a cloth.

Forced swimming test is a validated tool, previously described [11] to assess the depression-like behavior in rodents inside a transparent cylinder filled with water (water temperature 23–25°C) for 5 min. The level of water was adjusted to prevent the escape of the rats and importantly their tail could not touch the bottom of the cylinder.[12] The parameter, correlating with negative mood to measure, is the duration of the immobility, in which the rat merely floats to keep their head above the surface of the water. The immobility observed reflected a state of lowered mood or hopelessness in animals. [11, 13, 14]

Each rat was subjected to two swimming sessions separated by 24 h: the first trial lasted 15 min, whereas the second one, the only monitored, 5 min. Water was changed after every trial and animals were gently dried with a towel after swimming.

Statistical analyses

All data were expressed as mean ± SEM. Feeding data showed in Fig. 1A, left panel were statistically analyzed by three-way ANOVA for repeated measures, which included food restriction (no, yes) and stress (no, yes) as the between-subjects factors, with sessions time (0-15, 15-30, 30-60, 60-120 min) as the within-subject factor. Feeding data showed in Fig. 1B right panel, were statistically analyzed

by two-way ANOVA with food restriction and stress as the two factors. Feeding data showed in Fig. 1 (B-E) were statistically analyzed by one-way ANOVA with treatment as between-subject factor. Open field and forced swimming test (Table 2) were statistically analyzed by three-way ANOVA, which included food restriction (no, yes) and stress (no, yes) and treatment (no, yes) as the between-subjects factors. We used Bonferroni's post hoc tests to follow up on significant interaction or main effects ($P < 0.05$) from the factorial ANOVAs. We analyzed the data with Systat version 10.0 (Systat Software).

References

1. Bradford, M. M. (1976) A rapid and sensitive method for the quantitation of microgram quantities of protein utilizing the principle of protein-dye binding, *Anal. Biochem. 72*, 248-254.

2. Stoscheck, C. M. (1990) Quantitation of protein, *Methods Enzymol. 182*, 50-68.

3. DeHaven-Hudkins, D. L., Fleissner, L. C., and Ford-Rice, F. Y. (1992) Characterization of the binding of [3H](+)-pentazocine to sigma recognition sites in guinea pig brain, *Eur. J. Pharmacol. 227*, 371-378.

4. Battiti, F. O., Newman, A. H., and Bonifazi, A. (2020) Exception That Proves the Rule: Investigation of Privileged Stereochemistry in Designing Dopamine D3R Bitopic Agonists, *ACS Med. Chem. Lett., DOI: 10.1021/acsmedchemlett.9b00660*, in press.

5. Slack, R. D., Ku, T. C., Cao, J., Giancola, J. B., Bonifazi, A., Loland, C. J., Gadiano, A., Lam, J., Rais, R., Slusher, B. S., Coggiano, M., Tanda, G., and Newman, A. H. (2020) Structure–Activity Relationships for a Series of (Bis(4-fluorophenyl)methyl)sulfinyl Alkyl Alicyclic Amines at the Dopamine Transporter: Functionalizing the Terminal Nitrogen Affects Affinity, Selectivity, and Metabolic Stability, *J. Med. Chem. 63*, 2343-2357.

6. Cifani, C., Polidori, C., Melotto, S., Ciccocioppo, R., and Massi, M. (2009) A preclinical model of binge eating elicited by yo-yo dieting and stressful exposure to food: effect of sibutramine, fluoxetine, topiramate, and midazolam, *Psychopharmacology 204*, 113-125.

7. Bailey, K. R., and Crawley, J. N. (2009) Anxiety-Related Behaviors in Mice. In *Methods of Behavior Analysis in Neuroscience* (Buccafusco, J. J., Ed.), Boca Raton (FL), CRC press.

8. Alboni, S., Micioni Di Bonaventura, M. V., Benatti, C., Giusepponi, M. E., Brunello, N., and Cifani, C. (2017) Hypothalamic expression of inflammatory mediators in an animal model of binge eating, *Behav. Brain. Res. 320*, 420-430.

9. Micioni Di Bonaventura, M. V., Lutz, T. A., Romano, A., Pucci, M., Geary, N., Asarian, L., and Cifani, C. (2017) Estrogenic suppression of binge-like eating elicited by cyclic food restriction and frustrative-nonreward stress in female rats, *Int. J. Eat Disord. 50*, 624-635.

10. Thiel, C. M., Muller, C. P., Huston, J. P., and Schwarting, R. K. (1999) High versus low reactivity to a novel environment: behavioural, pharmacological and neurochemical assessments, *Neuroscience 93*, 243-251.

11. Porsolt, R. D., Anton, G., Blavet, N., and Jalfre, M. (1978) Behavioural despair in rats: a new model sensitive to antidepressant treatments, *Eur. J. Pharmacol. 47*, 379-391.

12. Gomes, F., Greidinger, M., Salviano, M., Couto, K. C., Scaperlli, G. F., Alves, S. H. d. S., and Cruz, A. P. d. M. (2010) Antidepressant- and anxiogenic-like effects of acute 5-HT2C receptor activation in rats exposed to the forced swim test and elevated plus maze, *Psychol. Neurosci. 3*, 245-249.

13. Porsolt, R. D., Bertin, A., and Jalfre, M. (1978) "Behavioural despair" in rats and mice: strain differences and the effects of imipramine, *Eur. J. Pharmacol. 51*, 291-294.

14. Porsolt, R. D., Le Pichon, M., and Jalfre, M. (1977) Depression: a new animal model sensitive to antidepressant treatments, *Nature 266*, 730-732.
